# Global Perspectives on Obesity and Being Overweight: A Bibliometric Analysis in Relation to Sustainable Development Goals

**DOI:** 10.3390/ijerph22020146

**Published:** 2025-01-22

**Authors:** Natália Ueda Yamaguchi, Letícia de Almeida, Rúbia Carvalho Gomes Corrêa, Rute Grossi Milani, Mirian Ueda Yamaguchi

**Affiliations:** 1 Department of Energy and Sustainability, Federal University of Santa Catarina, Campus Ararangua, Ararangua 88905-120, Brazil; 2 Center of Biological and Health Sciences, Cesumar University—UNICESUMAR, Maringa 87050-900, Brazil; almeidadeleticia@gmail.com; 3 Post-Graduation Program in Clean Technologies, Cesumar Institute of Science, Technology and Innovation, Cesumar University—UNICESUMAR, Maringa 87050-900, Brazil; rubia.correa@unicesumar.edu.br; 4 Post-Graduation Program in Health Promotion, Cesumar Institute of Science, Technology and Innovation, Cesumar University—UNICESUMAR, Maringa 87050-900, Brazil; rute.milani@unicesumar.edu.br (R.G.M.); mirian.yamaguchi@unicesumar.edu.br (M.U.Y.)

**Keywords:** agenda 2030, bibliometrics, health promotion, nutrition, SDG

## Abstract

Obesity and being overweight are significant risk factors for diseases and disabilities, making it crucial to address malnutrition in all its forms to ensure health and well-being for all, as well as to achieve sustainable development. This study conducted a bibliometric analysis of research on obesity in relation to Sustainable Development Goals (SDGs) using data from the Web of Science database from 2015 to 2024 and the VOSviewer software. The findings revealed that while research on obesity and SDGs has grown slowly, SDG 3 (Good Health and Well-Being) is predominant in the literature. This study highlighted the fragmentation of research due to the complex, multifactorial nature of obesity, emphasizing the need for a more holistic approach. Furthermore, international collaborations were found to be vital for advancing research and formulating effective public policies. This analysis also identified gaps in the research related to several SDGs, including education (SDG 4), affordable and clean energy (SDG 7), and partnerships (SDG 17), suggesting the need for a broader, more holistic approach. Additionally, emerging research related to SDG 11 (Sustainable Cities and Communities) underscores the importance of urban environments in tackling obesity. In conclusion, future research should adopt an interdisciplinary approach to address these gaps and contribute to advancing the 2030 Agenda.

## 1. Introduction

In 2015, the United Nations established the 2030 Agenda for Sustainable Development, an ambitious global framework aimed at promoting peace, prosperity, and well-being for people and the planet. The agenda includes 17 Sustainable Development Goals (SDGs) and 169 interlinked targets, intended to guide countries in addressing critical challenges to sustainable development. Among these, SDG 2 focuses on ending hunger, achieving food security, improving nutrition, and promoting sustainable agriculture. This goal incorporates targets to combat various forms of malnutrition, including undernutrition, being overweight, and obesity [1].

However, despite its inclusion under the broader category of malnutrition, obesity is not explicitly addressed in the targets of SDG 2, even though it poses a significant threat to global health and is interrelated with other SDGs, such as “Eradicating Poverty” (SDG 1) and “Health and Well-Being” (SDG 3). This oversight undermines the effectiveness of SDG 2 and other related goals, as obesity is a key driver of public health crises worldwide. According to recent studies, obesity is linked to a wide range of chronic, non-communicable diseases (NCDs), including hypertension, type 2 diabetes, cardiovascular disease, certain cancers, and even premature mortality [2,3,4]. Furthermore, it exacerbates social inequalities, affecting individuals’ mobility, employment opportunities, quality of life, and mental health [5].

The global rise in obesity has reached epidemic proportions. In 2022, 43% of adults worldwide were overweight, and 16% were obese—up from 25% of overweight adults in the 1990s. The global prevalence of obesity more than doubled between 1990 and 2022 [6]. Without effective interventions, the prevalence of overweight and obesity in children and adolescents (aged 5–19) is projected to rise from 430 million (22%) to 770 million (39%) by 2035 [7]. This trend highlights the urgency of addressing obesity as a critical component of the global health agenda.

Obesity results from a complex interplay of factors, including reduced physical activity, poor dietary patterns, socioeconomic disparities, and environmental influences. Lifestyle encompasses key pillars such as healthy eating practices, physical exercise, stress management, adequate sleep, and positive interpersonal relationships. Historical lifestyle changes that have encouraged the consumption of processed food diets and physical inactivity have been identified as major contributors to the global rise in being overweight and obesity [8,9]. In many low-income countries, rapid urbanization and shifts toward “Westernized” diets are contributing to rising obesity rates, while in high-income countries, lower-income populations often face greater exposure to unhealthy foods. Political stability, effective governance, and comprehensive public health policies are essential to addressing this issue at the global level [10]. Moreover, the global obesity crisis is compounded by climate-related challenges, which affect food security and access to healthy, affordable food.

Although the SDGs broadly acknowledge the need to reduce malnutrition, obesity often remains sidelined in global policy discussions. Given its profound impact on health outcomes and its role as a barrier to achieving the 2030 Agenda, a more focused approach is needed to incorporate obesity prevention and management into SDG frameworks.

In this context, bibliometric methodology emerges as a powerful tool for analyzing scientific production related to obesity and SDGs. Bibliometric analysis involves using statistical techniques to examine large volumes of scientific data to map research trends, identify knowledge gaps, assess the impact of publications, and guide decisions related to scientific policies and research investments [11]. In the case of obesity and the SDGs, bibliometrics allows researchers to identify how the topic has been addressed in the literature, which researchers and regions are most involved, and what action plans have been proposed to combat obesity as a challenge to sustainable development.

This study aims to apply bibliometric techniques to investigate how obesity has been addressed in the scientific literature related to SDGs, analyzing its impact on research and identifying emerging trends. The goal is not only to understand the current state of knowledge about obesity within the SDG framework but also to identify research gaps, guide future investigations, and provide insights for more effective public policies. In doing so, we aim to contribute to a clearer and more comprehensive understanding of how obesity can be more effectively integrated into global sustainable development strategies.

## 2. Materials and Methods

The scientific literature analysis was conducted through bibliometric analysis, following three phases, search and data collection, performance analysis and cluster analysis, and data visualization, according the methodology of previous studies [12]. The metadata used in this investigation were obtained from the core database of Clarivate Analytics’ Web of Science (WoS), part of the Institute for Scientific Information (ISI, Philadelphia, PA, USA). The WoS database was selected due to its coverage of high-impact journals and interdisciplinary areas, ensuring a broader and more diversified analysis. In contrast, PubMed is specific to the biomedical field, which could bias the results by focusing predominantly on SDG 3, thereby limiting the representation of other SDGs. Article-type documents were retrieved using the search keywords (“obesity*” or “overweight*”) AND (“sustainable development” or “sdg*” or “agenda 2030”) in the TOPIC field within the expanded SCI collection. The search was performed on 28 May 2024 and was limited to English-language articles published after 2015, resulting in 296 documents. Each document was independently screened by a minimum of two authors to verify if it met the inclusion criteria, narrowing the selection to 161 documents. The documents were analyzed using the VosViewer software (version 1.6.18) according to the number of documents, research areas, SDGs, most productive sources, most cited documents, bibliographic coupling, most productive countries, co-authorship analysis, and keyword co-occurrence. An interpretative analysis [13,14] was used to explore the conceptual framework of the research field and identify thematic streams.

## 3. Results

### 3.1. Number of Documents

**Figure 1:**
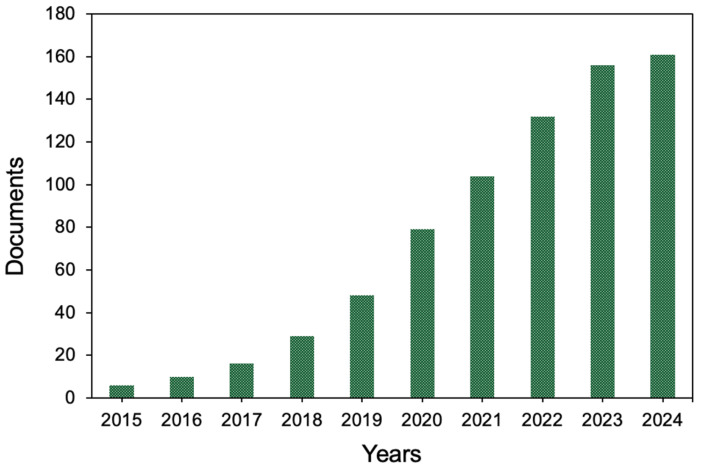
Cumulative scientific production of documents on being overweight, obesity, and Sustainable Development Goals.

Figure 1 illustrates the cumulative distribution of scientific publications on obesity, being overweight, and SDGs from 2015 to May 2024. The growth in the number of publications is uneven over time. In 2015, only one document was published on the topic, and publication rates increased gradually in the following years. However, it was only after 2019 that the annual number of articles began to surge, tripling in volume. In 2020, a record of 31 articles were published, marking the most productive year in terms of research on this issue. Since then, the number of publications has continued to rise at a modest pace, reaching a total of 161 publications by May 2024.

### 3.2. Most Productive Sources

Table 1 presents the journals with the highest citation counts. The journal with the highest number of citations was *Food Security*. Most of the journals are focused on food, nutrition, and agriculture, with a smaller proportion representing public health and broader sustainability topics. Notably, only one journal specializing in obesity appeared among the most cited.

Regarding the number of documents (D) per journal, the journal *Sustainability* published the highest number of articles. The average citations per document (ACs) indicate that the journals Science of the Total Environment, Food Security, and the European Journal of Public Health stand out. However, when considering the normalized citation (NC), which is the number of citations of a document divided by the average number of citations of all documents published in the same year included in the dataset, it was found that Science of the Total Environment and Sustainability were the most prominent journals. This normalization corrects the fact that older documents have more time to accumulate citations than more recent ones [14].

However, the average normalized citation (ANC), which represents the NC divided by the number of documents published by each journal, revealed that Science of the Total Environment, the European Journal of Public Health, and Global Food Security–Agriculture, Policy, Economics, and Environment were the most notable journals. It is important to emphasize that the number of citations alone is not a sufficient measure for evaluating the relevance of a document or journal [15].

### 3.3. Most Cited Documents

Table 2 presents the most cited documents in the research on obesity and the SDGs, highlighting the most influential publications in the field. These documents were selected based on the number of citations received, reflecting their impact and relevance in the academic literature. The analysis of these articles provides an in-depth view of the main contributions to understanding the interactions between obesity and the SDGs, as well as the approaches most widely discussed in the global context.

#### Bibliographic Coupling

**Figure 2:**
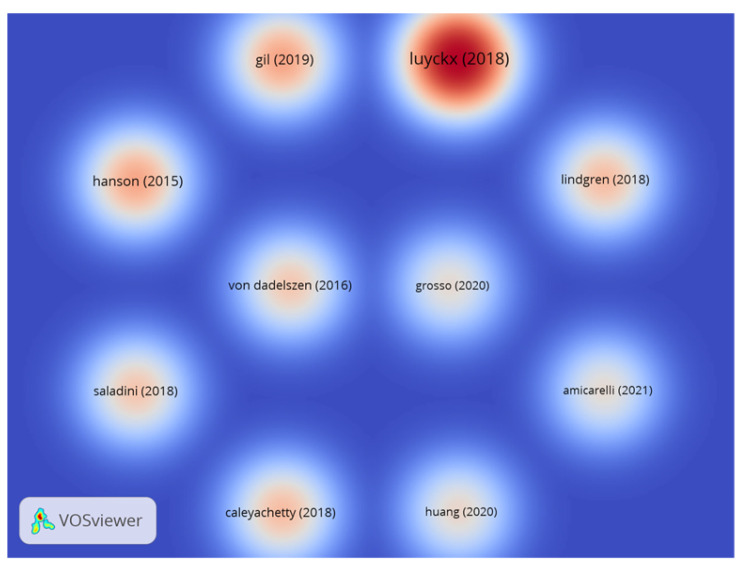
A bibliographic coupling density diagram of the 10 most cited documents (minimum number of citations per document: 62, threshold met: 10, clusters: 10, links: 0, weights: citations, VosViewer 1.6.18).

Furthermore, an analysis of the bibliographic coupling density of the ten most cited documents is presented in Figure 2. To conduct this analysis, a citation threshold of 62 citations per document was applied to select the ten most cited articles. As a result, ten clusters were formed, with no inter-document links identified, where the citation count served as the weight for each document. It is evident that the documents did not exhibit bibliographic coupling, as the number of citations is represented by the intensity of the red color, while the proximity of the documents would indicate potential bibliographic coupling.

### 3.4. Most Productive Countries

**Figure 3:**
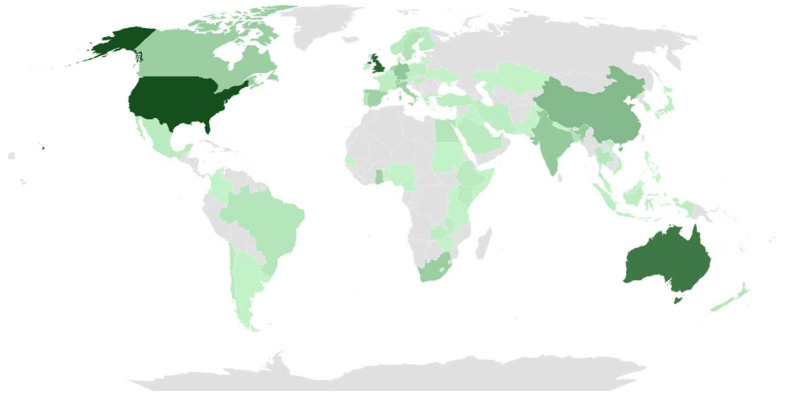
Geographic distribution of documents.

To represent the significance of document production by the most productive countries based on the affiliations of the corresponding authors, a total of 78 countries were included (Figure 3). The United States was the most prolific country, accounting for 25% of the documents (41), followed by England and Australia, which represented 21% (34) and 19% (31), respectively. Lastly, China contributed 10.9% (15) of the scientific production, followed by Germany and India, each with 7%.

#### Co-Authorship Analysis

**Figure 4:**
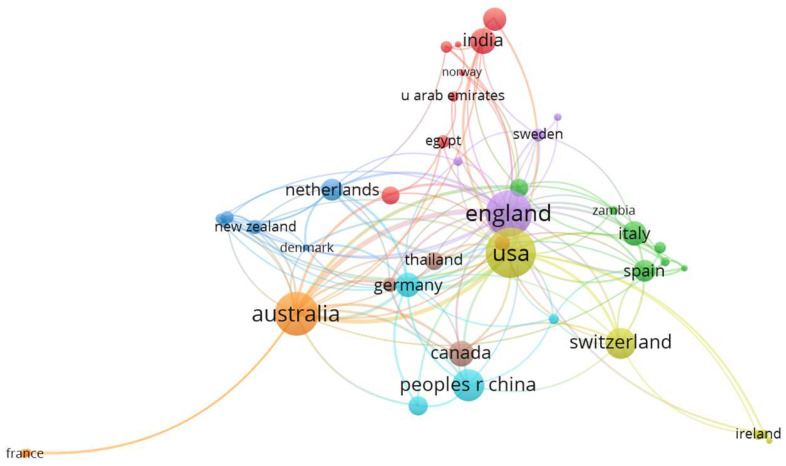
A map created based on country co-authorship according to author affiliations (minimum number of documents per country: 2; items meeting the threshold, the total of 78 countries: 46; clusters: 8; links: 150; total link strength: 257; weights: normalized citations; VOSviewer 1.6.18.).

To better understand international collaborations, a mapping of country co-authorship based on author affiliations is presented in Figure 4. All countries with at least two publications were included. This condition was met by 46 countries, resulting in eight clusters, 150 links, and a total link strength of 257. The size of the circle reflects the number of normalized citations (weights) associated with each country in the dataset. Only 41 countries had links, with Iraq, Poland, Greece, Ethiopia, and Hungary showing no co-authorship with other countries.

### 3.5. Research Areas

**Figure 5:**
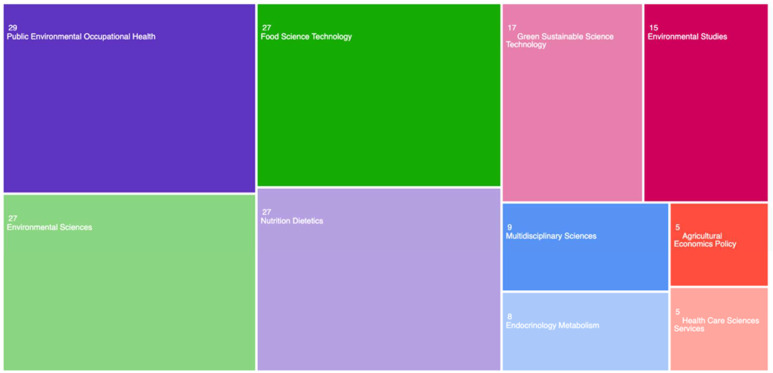
Documents classified by thematic category in the Web of Science.

The thematic category with the highest number of publications in the WoS was public environmental occupational health, which accounted for 18.0% of the total documents analyzed (Figure 5). This was followed by environmental sciences, food science technology, and nutrition dietetics, each contributing 16.8% of the total publications. Other notable categories included green sustainable science technology (10.6%) and environmental studies (9.3%), which also represented a significant portion of the total documents. It is important to note that publications can appear in multiple categories. Overall, the categories were primarily concentrated in the fields of health, food, and sustainability, reflecting the keywords used in the research, which included terms such as being overweight, obesity, and sustainable development.

### 3.6. SDGs

**Figure 6:**
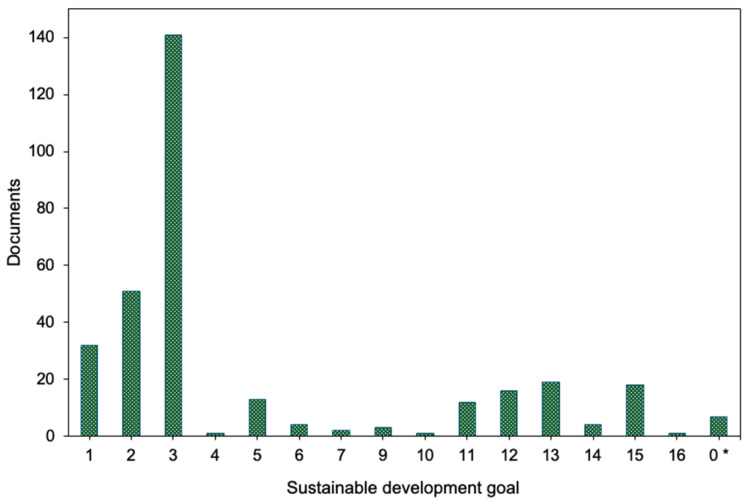
The distribution of publications according to the Sustainable Development Goals (SDGs) addressed (* documents not classified according to the SDGs).

The documents were classified according to the relevant SDG using the mapping tool available on the WoS platform. The results are presented in Figure 6. It was observed that SDG 3 dominates significantly, accounting for 88% of the total documents. This result is consistent with the previous finding, where public, environmental, and occupational health emerged as the most broadly represented theme, causing the prominence of SDG 3.

The documents were primarily linked to all the SDGs, except for SDGs 8 and 17. Seven documents were not associated with any SDG. It is important to emphasize that the SDGs are interdependent, indivisible, and should be addressed in a balanced manner across the three dimensions of sustainable development: economic, social, and environmental. Therefore, achieving these goals is not feasible without ensuring a balance between these dimensions [1].

### 3.7. Keywords Co-Occurrence

**Figure 7:**
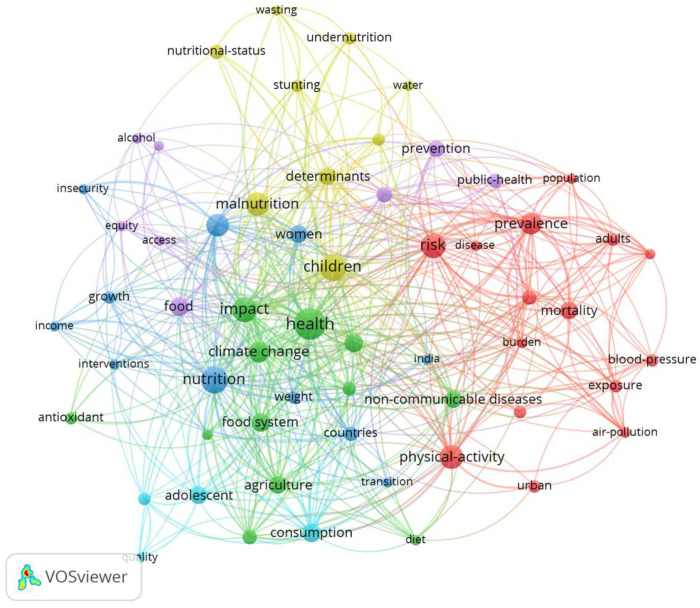
Clusters based on the co-occurrence of all keywords (minimum number of occurrences per keyword: 4; items meeting the threshold: 59; clusters: 6; links: 588; total link strength: 950; weights: occurrences, VosViewer 1.6.18).

Keyword occurrence was used to investigate how research on being overweight, obesity, and sustainable development has been structured. Figure 7 and Table 3 present the results of the co-occurrence analysis of 1046 keywords from 161 analyzed documents. The minimum number of occurrences for a keyword was set to four, and no minimum citation threshold for the documents was required. The resulting map comprised 59 keywords and six clusters, with 588 links and a total link strength of 950. The size of each circle reflects the number of occurrences (weights) of each keyword within the dataset.

## 4. Discussion

The increasing global prevalence of obesity, its significant implications for public health, and the substantial challenges associated with its prevention and treatment make obesity one of the most critical public health issues worldwide [26]. Despite growing interest from the scientific community on this topic, there has been only a modest increase in the number of academic publications examining the intersection between obesity, being overweight, and the SDGs in recent years (Figure 1).

This limited academic attention can be attributed to the historical neglect of obesity in previous development frameworks. Nearly a decade after the publication of the 2030 Agenda, the lack of formal recognition of obesity in the SDGs remains a significant barrier, hindering the effective implementation of interventions aimed at addressing this global health challenge [10]. As a result, both international health funders and policymakers often overlook this critical issue. Consequently, the connection between obesity and the SDGs has been undervalued and insufficiently explored, leading to a relatively scarce body of literature on the subject [10].

Upon examining the most cited journals (Table 1), it is evident that research addressing sustainability, public health, and food security holds a prominent position, underscoring the comprehensive and interdisciplinary nature of the topics of obesity and being overweight within the context of the SDGs.

Regarding the most cited documents (Table 2), a significant diversity in the central research themes was observed, emphasizing the multifactorial and complex nature of the issue of obesity. This characteristic is further corroborated by Figure 4, which illustrates the bibliographic decoupling between the most cited documents.

Among the most cited documents, two focused primarily on diseases related to SDG 3, specifically hypertensive disorders [22] and chronic kidney disease [16], with obesity addressed as a secondary issue. Other researchers have explored various factors contributing to the obesity epidemic, including environmental and nutritional factors that influence obesity risk [18], as well as the role of urban green areas in mitigating obesity by promoting physical activity [23].

The other most cited documents cover a range of topics associated with various SDGs. These include the following: sustainable food systems [20], related to SDG 12; the water-energy-food nexus [21], linked to SDG 6; food waste [24], which connects to both SDGs 12 and 2; nutrition and its connection to SDG 2 [25]; malnutrition among adolescents in low- and middle-income countries [19], relevant to SDGs 1, 2, 3, and 10; and a review of the indicators for SDG 2 [17].

The results regarding the most productive countries revealed significant variation in the global distribution of scientific production on obesity, being overweight, and the SDGs (Figure 3). This variation can be attributed to differences in research incentives, the available infrastructure, and the funding levels in each country. Additionally, this discrepancy may reflect factors such as population size and specific research priorities, which vary according to each nation’s political, social, and economic contexts. For example, in the United States, obesity rates have more than tripled since the 1960s. As a result, a variety of approaches to addressing obesity have been adopted, including the proposal and implementation of public policies that affect multiple environments. This reflects increasing federal awareness and concern, which has driven greater investments and a significant rise in research production in this area [27].

Co-authorship in academic research, as illustrated in Figure 4, plays a pivotal role in fostering global scientific collaboration, enhancing both the quality and impact of research outputs. Studies have shown that international collaborations tend to result in higher citation rates, indicating that such work has a broader academic reach compared to national collaborations alone [28]. A notable example is the highly cited article “The Global Burden of Kidney Disease and the Sustainable Development Goals” [16] co-authored by researchers from Switzerland, Canada, and the United States. This internationalization of data on obesity and the SDGs is crucial for understanding both global and regional trends, evaluating economic impacts, and shaping effective public policies. Given the time-consuming and costly nature of data collection, international collaboration becomes indispensable in assessing and addressing these challenges [29,30].

Moreover, such collaborations allow for a deeper understanding of regional disparities in obesity and SDG-related issues, with insights that are critical for tailoring interventions to the specific geographical, demographic, and socioeconomic contexts of different regions. Identifying global and regional patterns, fostering international cooperation, disseminating effective strategies, and guiding future research are essential steps in both the prevention and treatment of obesity and in achieving the broader objectives of the 2030 Agenda. These efforts will ultimately be key to addressing the complex, multifaceted nature of obesity and its intersection with the SDGs [29,30].

Among the areas of research analyzed (Figure 2), the area of “Public, Environmental, and Occupational Health” stood out as the most relevant in terms of publication volume. This result is justifiable, as obesity and being overweight are conditions determined by a series of interconnected factors, including individual behaviors, social influences, environmental conditions, and occupational factors [31], which directly impact lifestyle choices, access to healthy foods, and physical activity, and, consequently, have significant implications for public, environmental, and occupational health [32].

Environmental and occupational health are fundamental subfields in research, public policy formulation, and public health practice [33]. It is now recognized that the health of a society is largely shaped by its economic system and the development process, as environmental health is directly influenced by transformations caused by the anthropogenic environment [34]. Economic development, coupled with urbanization, natural resource extraction, industrialization, and the transportation systems supporting this model, significantly alters the natural environment, often compromising the integrity of ecosystems [35].

The classification of the documents according to the SDGs revealed that the majority were assigned to SDG 3. It is important to note that, although obesity is commonly addressed under target 2.2 of SDG 2, which aims to eliminate all forms of malnutrition, this study reveals that SDG 2 was categorized in only 32% of the analyzed documents, while SDG 3 was associated with 88%. This finding suggests a potential gap in the formulation of the Agenda 2030 targets, as obesity is not recognized as a priority issue under SDG 2 or 3, resulting in its treatment as a condition separate from the nutrition-related target.

The other SDGs associated were SDG 1 (20%), SDG 13 (12%), SDG 11 (11%), and SDG 12 (10%). This result aligns with the findings from the research areas, the most cited articles, and the most cited journals, indicating that the main topics addressed are related to health, nutrition and agriculture, social issues, and sustainability.

The co-occurrence map of keywords from the articles generated a large cluster (Figure 7), which, although divided into six main clusters, shows a strong interconnection among them, indicating that all keywords are related.

The first cluster (red) is primarily associated with the core theme of diseases caused by obesity and being overweight, such as mortality, hypertension, high blood pressure, and pregnancy complications. This group also includes physical activity and body mass index. It is well established that obesity can be prevented through physical activity, as it promotes a negative energy balance by increasing caloric expenditure, improving overall health, preventing weight regain after initial weight loss, maintaining lean mass, and thereby enhancing body composition [36].

In cluster 1, the keyword air pollution stood out with the highest ANC (2.29). Several studies indicate a positive association between exposure to air pollutants and an increase in body mass index, as well as the development of obesity. The underlying mechanisms of this relationship may be linked to the fact that poor air quality contributes to increased oxidative stress, inflammation, and the risk of chronic diseases, as well as discouraging outdoor physical activity—factors that promote sedentary behavior and weight gain [37,38,39]. However, other studies found contrary results, where rural areas showed a stronger association with obesity compared to urban areas. This phenomenon may be explained by factors such as higher socioeconomic vulnerability, less healthy dietary habits, and reduced access to healthcare, characteristics that make rural populations more susceptible to the effects of pollution on obesity [40,41]. Therefore, this cluster may be associated with SDGs 2, 3, and 11.

In the second cluster, the keyword health appears with the highest number of occurrences (34), but it is associated with a relatively low ANC (0.85). As previously noted, most of the documents are linked to the health research area, aligning with SDG 3. However, this cluster predominantly emphasizes environmental impacts and climate change, thus highlighting SDG 13. It also draws attention to agriculture (SDG 2) and sustainable production practices (SDG 12). Agriculture is a significant contributor to global warming and climate change, primarily through the emission of greenhouse gases (GHGs) resulting from activities such as deforestation for agricultural expansion, food production, fertilizer use, and waste management [42]. Climate change, in turn, directly threatens food security, agricultural productivity, and even human health [43]. In this context, several studies explore mitigation strategies, including diets with reduced GHGs [42,44,45], sustainable food policies and systems [46,47], and alternative protein sources such as edible insects [48].

The third cluster addresses topics such as food security, interventions, and women, thus focusing on vulnerable populations and low-income countries. Specifically, this cluster discusses obesity in the context of social factors such as poverty, public health, gender equality, and a reduction in inequalities, reflecting SDGs 1, 3, 5, and 10, respectively. The key topics explored include socioeconomic and demographic determinants related to obesity [49,50,51], being overweight and obesity during pregnancy [50,52]; obesity and gender-based violence [53], and food justice and equity [32,54].

The keywords domestic, determinants, undernutrition, malnutrition, and water showed the highest ANC in Cluster 4. Thus, in addition to SDG 2, which addresses undernutrition and malnutrition, SDG 6 is also represented in this cluster. The studies related to water include topics such as lower water consumption linked to reduced obesity, given the lower need for food production [55], algae with potential properties to combat obesity [56], obesity as a positive index for measuring the water–energy–food nexus [21], and substances in water that can disrupt the endocrine system [57]. Furthermore, the keyword children is included in this cluster. Many studies were found that focused on children as a vulnerable population. One study found a higher likelihood of being overweight/obesity among school-age children with a diverse diet. Although there is a variety of food groups available, many children predominantly consume calorie-dense foods, with limited intake from other essential food groups. This dietary imbalance contributes to a higher risk of being overweight and obesity. Furthermore, a decrease in physical activity among school-aged children may intensify their susceptibility to these conditions [58].

Furthermore, studies demonstrate the significant influence of parents on childhood obesity, encompassing aspects ranging from eating habits and lifestyle choices to socioeconomic factors. Healthy eating behaviors by parents can reduce the risk of obesity in their children, even among those with a genetic predisposition, although a balanced diet alone does not completely neutralize the heritability of obesity [59]. Furthermore, factors related to parental lifestyle, from the preconception period to pregnancy, are associated with a higher risk of obesity in children, particularly a high body mass index, smoking, poor diet quality, and sedentary behavior [60]. Similarly, the parental education level also influences this risk: the higher the level of education, the lower the chances of children developing obesity or being overweight [61,62].

The keywords in the fifth cluster encompass public health, alcohol, and tobacco. Non-communicable diseases are often aggravated by smoking, alcohol consumption, and obesity, leading several studies to address these factors in conjunction [63]. Consequently, many preventive measures aim to tackle these issues simultaneously. This cluster is clearly delineated, with its themes primarily aligned with SDGs 2, 3, and 10. Furthermore, equity emerges as a central concern, with SDGs 1, 2, 5, and 10 being closely intertwined as they target vulnerable and underserved populations.

Inequality prevents certain individuals from accessing healthy foods and diets, as well as other factors necessary to ensure proper nutrition. Inequality in malnutrition results from unjust structural processes driven by the unequal distribution of power and social exclusion [32]. A common feature of food environments in many low- and middle-income countries is the growing prevalence of processed foods that are low in nutritional value yet are conveniently available, economically affordable, and highly desirable [64].

Finally, the sixth cluster includes the keywords quality, consumption, adolescents, and diet. Most articles associated with these keywords primarily focus on adolescent girls [65,66], thereby aligning with SDG 5.

The articles in this study cover a wide range of topics that contribute to the SDGs, fostering innovations in various fields. The aim of this article was to understand research trends to guide new studies, assess economic impacts, and support the formulation of effective public policies. However, it was observed that some areas remain underexplored, and certain SDGs are still neglected. Thus, Table 4 was created to illustrate the themes identified in the analyzed articles, as well as the interrelationships, contributions, and causal links between each SDG and the conditions of obesity and being overweight.

Obesity is a complex, multifactorial condition that intersects with all SDGs, each addressing different aspects of its causes, consequences, and potential solutions. Poverty (SDG 1) restricts access to healthy foods, due to factors like cost, lack of storage, or inadequate nutrition monitoring, leading to poor dietary habits. Unsustainable food production (SDG 2) exacerbates undernourishment, while optimizing diets and improving food production are crucial for combating both undernutrition and obesity.

Obesity, linked to non-communicable diseases (SDG 3), is a leading cause of premature death, making its prevention and treatment essential. Promoting healthy eating habits (SDG 2) and physical activity is key to addressing this issue. Education (SDG 4) is vital in raising awareness about nutrition, the importance of exercise, and healthy lifestyle choices. A lack of proper nutrition education contributes to higher obesity rates, as many individuals are not informed about the health consequences of poor dietary choices. Furthermore, education represents an opportunity to combat the issue of transgenerational repercussions of obesity. Pedagogical intervention measures targeting lifestyle have shown positive results in preventing and treating obesity when applied in early childhood and higher education. Additionally, the educational level of parents has been shown to influence these habits, consequently impacting their children’s health [67,68].

Gender equality (SDG 5) is impacted by obesity as it affects women’s health, self-esteem, economic opportunities, and access to healthcare while increasing stigma and discrimination. Environmental factors, such as endocrine-disrupting chemicals in water (SDG 6) and water consumption linked to increased food production (SDG 6), also contribute to obesity. Clean and affordable energy (SDG 7) is essential to support sustainable food systems, from production to storage.

Decent work (SDG 8) allows individuals to have the time and resources necessary for healthy living, while sustainable cities (SDG 11) promote environments conducive to physical activity and healthy eating. Food science technologies (SDG 9) are necessary for the development of nutritious and affordable foods, while urban planning (SDG 11) plays a key role in reducing obesity by promoting walkable cities and access to fresh foods.

Socioeconomic disparities (SDG 10) challenge access to healthy food and active living, with unequal access to healthy environments contributing to higher obesity rates. Climate change (SDG 13) affects food availability and prices, increasing reliance on unhealthy, processed foods, while sustainable agriculture and aquaculture (SDG 14, SDG 15) offer pathways to better nutrition. Lastly, inclusive societies (SDG 16) and partnerships (SDG 17) foster collaborative efforts to tackle obesity and its social determinants.

The results clearly demonstrate that emphasizing lifestyle as a fundamental strategy to combat being overweight and obesity remains underrepresented in global perspectives. While genetic, environmental, and socioeconomic factors are undoubtedly relevant, lifestyle continues to be the most actionable and impactful factor in addressing these issues. An integrated approach that prioritizes lifestyle changes while addressing broader influences is essential to achieving sustainable outcomes.

The global perspective on obesity often highlights the need for multidimensional strategies, incorporating lifestyle modifications alongside public policies, health system interventions, and environmental changes. However, this integrated approach tends to diminish the prominence of lifestyle changes as a standalone solution. Lifestyle modifications should instead be positioned as a significant component of a broader solution, complementing policy and medical interventions aimed at promoting health, preventing obesity and being overweight, and, consequently, mitigating the risk of other diseases.

The findings of the current article reveal that lifestyle has been regarded as secondary to other determinants concerning the Sustainable Development Goals (SDGs). Nevertheless, substantial evidence in the literature underscores the pivotal role of lifestyle in combating obesity through its influence on dietary habits, physical activity, and behavioral patterns. Elevating the significance of lifestyle represents one of the critical gaps observed in this study, especially in the context of the 2030 Agenda and the global fight against being overweight and obesity.

Addressing obesity requires a comprehensive and integrated approach involving health, education, environmental, and social factors, aligned with multiple SDGs. This holistic strategy is essential to create sustainable, equitable, and health-promoting systems capable of reducing the global burden of obesity.

### Limitations, Strengths, and Future Research

The bibliometric analysis conducted in this study provides a detailed understanding of the development of research on obesity in the context of the Sustainable Development Goals (SDGs), identifying emerging trends, challenges, and underexplored areas. The main limitations, strengths, and opportunities for future investigations are as follows:

One of the primary limitations identified is the slow growth in scientific output related to being overweight, obesity, and the SDGs, reflecting the complexity of the issue and the lack of clearly defined goals for addressing obesity within the SDG framework. Additionally, the analysis focused exclusively on the Web of Science platform, which may have limited the availability of data by excluding relevant information from other databases, such as Scopus or PubMed. Another significant limitation is the predominantly quantitative nature of the study, which excluded a qualitative analysis that could provide deeper insights, such as the positive and negative impacts of interventions adopted for the prevention and treatment of obesity and being overweight. It is important to note that the high-impact articles identified in this analysis, based on citation counts, do not always reflect the intrinsic quality of the studies, as these indicators can be influenced by collaboration networks, self-citations, and publishing trends during specific periods. Lastly, the frequent absence of the term “lifestyle” in the keywords of articles limits the exploration of its critical role as a central factor in combating obesity. The lack of proper keyword indexing is a significant drawback, as it is essential for enhancing accessibility, academic impact, and guiding future research toward more effective strategies in bibliometric analyses.

Despite these limitations, the study highlights important strengths. The concentration of publications on SDG 3 (Good Health and Well-Being) demonstrates the significant emphasis on health-related aspects, while other areas, such as nutrition, social issues, and sustainability, also received notable attention. The analysis showed that international collaborations are crucial for enhancing the impact of scientific research, promoting a broader global understanding, and contributing to more effective public policies. Furthermore, the study identified the most productive countries, such as the USA, the UK, Australia, and China, highlighting disparities in research funding and infrastructure among nations.

The bibliometric analysis identified imbalances in the exploration of certain SDGs. Goals such as SDG 4 (Quality Education), 7 (Affordable and Clean Energy), 8 (Decent Work and Economic Growth), and 17 (Partnerships for the Goals) were less explored, emphasizing the need for a more integrated approach that connects obesity to other targets within the 2030 Agenda. Keywords like “air pollution” and “urban,” frequently mentioned in the literature, reveal the relevance of environmental factors in developing sustainable solutions, particularly within the scope of SDG 11 (Sustainable Cities and Communities).

Therefore, future research could prioritize qualitative analyses to better understand the impacts of interventions, expand the use of databases to ensure greater coverage, and address underexplored SDGs with an interdisciplinary perspective, promoting a holistic and integrated approach to studying obesity in the context of sustainable development.

## 5. Conclusions

In conclusion, obesity is a multifaceted and complex global challenge that intersects with various SDGs, highlighting its intricate causes and far-reaching consequences. This study has demonstrated that, despite the growing recognition of obesity’s impact on public health, there remains a significant gap in the academic literature exploring its relationship with the SDGs. The lack of formal acknowledgment within previous development frameworks, particularly the 2030 Agenda, hampers the effective implementation of interventions and limits the resources allocated to combating obesity.

Addressing obesity requires a comprehensive and integrated approach, prioritizing lifestyle modifications while recognizing the critical role of public policies, health interventions, education, and environmental factors. Evidence underscores that, although genetic, environmental, and socioeconomic influences are relevant, lifestyle changes—including healthier dietary habits, increased physical activity, and behavioral shifts—are among the most effective and impactful strategies. Policies that elevate the role of lifestyle within global perspectives and align these efforts with the 2030 Agenda are essential to effectively combat obesity. By fostering global collaboration across sectors and integrating lifestyle-focused strategies with broader structural interventions, sustainable and equitable systems can be built that promote health, prevent obesity, and mitigate its cascading effects on society. Such a holistic strategy is crucial to reducing the global burden of obesity and advancing progress across multiple SDGs.

Finally, this study calls for broader recognition of obesity within the SDG framework, encouraging the development of holistic strategies that address the root causes of obesity, promote healthier lifestyles, and support sustainable public health policies. To effectively tackle the global obesity epidemic and its far-reaching consequences, there is an urgent need for more targeted research, stronger international cooperation, and a commitment to ensuring equitable access to the resources and environments necessary for healthier lives.

## Figures and Tables

**Table 1:** The most cited sources.

Rank	Source	TC	D	AC	NC	ANC	IF	Cite Score	h-Index	Publisher
1	*Food Security*	152	6	25	5.3	0.89	5.6	14	76	Springer Nature (London, England)
2	*Sustainability*	140	11	13	6.4	0.58	3.3	6.8	169	MDPI (Basel, Switzerland)
3	*Global Food Security*	130	4	33	5.4	1.36	9.8	20.9	81	Elsevier (Amsterdam, The Netherlands)
4	*Science of the Total Environment*	103	4	26	6.8	1.69	8.2	17.2	353	Elsevier (Amsterdam, The Netherlands)
5	*European Journal of Public Health*	72	3	24	4.0	1.34	3.7	5.6	111	Oxford University Press (Oxford, London)
6	*Bmj Global Health*	64	3	21	2.3	0.77	7.1	11.4	76	Bmj (London, England)
7	*Plos One*	56	4	9	4.4	0.73	2.9	6.2	435	Public Library of Science (San Francisco, CA, USA)
8	*Current Developments in Nutrition*	56	6	14	2.5	0.61	3.8	5.3	34	Elsevier (Amsterdam, The Netherlands)
9	*Current Obesity Reports*	53	5	11	2.1	0.41	9.5	16.4	66	Springer Nature (London, England)
10	*Nutrients*	152	5	10	3.0	0.59	4.8	9.2	209	MDPI (Basel, Switzerland)

D, number of documents; TC, total citations; AC, average number of citations per document; NC, normalized citations; ANC, average normalized citations; h-index, Hirsch index; IF, impact factor.

**Table 2:** The most cited documents.

Rank	Source	Journal	TC	NC	Ref.
1	The global burden of kidney disease and the sustainable development goals	*Bulletin of the World Health Organization*	443	6.3	[16]
2	Sustainable development goal 2: Improved targets and indicators for agriculture and food security	*Ambio*	129	4.8	[17]
3	Developmental origins of health and disease–Global public health implications	*Best Practice & Research Clinical Obstetrics and Gynaecology*	126	3.0	[18]
4	The double burden of malnutrition among adolescents: analysis of data from the Global School-Based Student Health and Health Behavior in School-Aged Children surveys in 57 low- and middle-income countries	*The American Journal of Clinical Nutrition*	99	1.4	[19]
5	Sustainable food systems—a health perspective	*Sustainable Science*	98	1.4	[20]
6	Linking the water-energy-food nexus and sustainable development indicators for the Mediterranean region	*Ecological Indicators*	88	1.2	[21]
7	Preventing deaths due to the hypertensive disorders of pregnancy	*Best Practice & Research Clinical Obstetrics and Gynaecology*	86	2.3	[22]
8	Association between community greenness and obesity in urban-dwelling Chinese adults	*Science of the Total Environment*	67	3.7	[23]
9	Food waste measurement toward a fair, healthy and environmental-friendly food system: a critical review	*British Food Journal*	62	4.2	[24]
10	Nutrition in the context of the Sustainable Development Goals	*European Journal of Public Health*	62	3.5	[25]

TC, Total citations; NC, normalized citations; Ref, references.

**Table 3:** The general characteristics of the clusters based on keyword co-occurrence.

Cluster	Keywords *	Items	ANC	AC	Occurrences
1 (red)	Air-pollution, exposure, risk, hypertension, pregnancy, blood-pressure, adults, urban, body-mass index, prevalence, burden, disease, physical-activity, mortality, population.	15	1.1 ± 0.2	20.3 ± 7.1	7.3 ± 4.9
2 (green)	Antioxidant, policy, agriculture, environment, climate change, impact, non-communicable diseases, food system, health, security, diet, nutrition transition.	12	1.1 ± 0.4	21.0 ± 11.7	11.3 ± 8.5
3 (blue)	Income, India, interventions, nutrition, food security, transition, growth, countries, women, weight, insecurity.	11	1.1 ± 0.5	17.3 ± 7.1	7.9 ± 6.4
4 (yellow)	Household, determinants, undernutrition, water, malnutrition, wasting, children, stunting, nutritional-status.	9	1.0 ± 0.3	17.4 ± 15.8	9.2 ± 7.3
5 (purple)	Access, prevention, equity, food, public-health, alcohol, tobacco, association	8	1.2 ± 0.6	34.1 ± 37.8	6.4 ± 2.8
6 (cyano)	Quality, consumption, adolescent, mediterranean diet.	4	0.7 ± 0.2	12.0 ± 6.9	7.5 ± 3.5

AC, average citation; ANC, average normalized citation. * Descending order of ANC.

**Table 4:** Being overweight and obesity in the context of the Sustainable Development Goals (SDGs).

SDG	Relationships, Contributions, and Causal Links
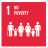	Poverty restricts access to adequate nutrition, either due to the cost of healthy foods, insufficient storage facilities, or the difficulties in adhering to nutritional recommendations and monitoring nutritional status.
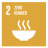	Unsustainable food production leads to undernourishment, while optimizing dietary patterns and providing more nutritious foods are essential to reversing this situation.
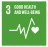	Obesity is a growing condition linked to several non-communicable diseases that can cause premature death. Combating obesity is crucial, and it involves health promotion, prevention, and treatment.
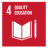	The promotion of healthy eating habits and awareness of the importance of physical activity can affect healthy and sustainable food choices.
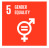	Obesity affects gender equality and women’s empowerment, influencing their health, self-esteem, access to healthcare, and economic opportunities, while also exacerbating stigma and discrimination.
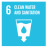	Endocrine-disrupting chemicals in water can pose health risks that contribute to obesity. Moreover, water consumption can be associated with obesity, as greater food intake drives increased food production.
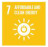	Clean and affordable energy is important for food, cooking, production, transportation, processing, storage, and ensuring food security.
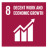	Decent jobs and fair working conditions provide more time and resources for healthy eating and physical activity, while also creating environments that promote workers’ health and well-being.
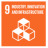	Food science technologies and affordable access to infrastructure are essential for the development of new processes and affordable healthy foods.
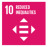	Socioeconomic disparities lead to increased challenges in accessing healthy foods and maintaining active lifestyles, factors that significantly contribute to the prevalence of being overweight and obesity.
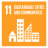	Healthy and sustainable urban environments, which offer access to fresh foods and opportunities for physical activity, are crucial for preventing obesity and promoting healthy lifestyles.
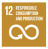	The production of healthy foods, responsible consumption, and sustainability in the food supply chain are essential to combating obesity and promoting more nutritious and sustainable food systems.
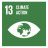	Climate change affects food availability and prices, increasing reliance on unhealthy, processed foods, while plant-based diets offer benefits for both health and greenhouse gas mitigation.
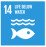	Aquaculture enhances nutrition by providing sustainable protein sources, but overfishing threatens marine biodiversity and undermines long-term sustainability.
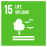	Unsustainable agricultural practices can compromise the production of nutritious food, exacerbating food insecurity and the factors that contribute to being overweight and obesity.
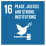	Inclusive societies, with equitable access to justice and peace, can contribute to obesity prevention by ensuring effective public policies and creating healthy environments for vulnerable populations.
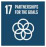	Partnerships can combat obesity by facilitating resource sharing, knowledge exchange, and strengthening collaboration across various involved sectors and governments.

## Data Availability

Raw data are available upon request.
